# Chronic fatigue syndrome: identifying zebras amongst the horses

**DOI:** 10.1186/1741-7015-7-58

**Published:** 2009-10-12

**Authors:** Samuel B Harvey, Simon Wessely

**Affiliations:** 1 Institute of Psychiatry, King's College London, London, UK

## Abstract

There are currently no investigative tools or physical signs that can confirm or refute the presence of chronic fatigue syndrome (CFS). As a result, clinicians must decide how long to keep looking for alternative explanations for fatigue before settling on a diagnosis of CFS. Too little investigation risks serious or easily treatable causes of fatigue being overlooked, whilst too many increases the risk of iatrogenic harm and reduces the opportunity for early focused treatment. A paper by Jones *et al *published this month in *BMC Medicine *may help clinicians in deciding how to undertake such investigations. Their results suggest that if clinicians look for common psychiatric and medical conditions in those complaining of prolonged fatigue, the rate of detection will be higher than previously estimated. The most common co-morbid condition identified was depression, suggesting a simple mental state examination remains the most productive single investigation in any new person presenting with unexplained fatigue. Currently, most diagnostic criteria advice CFS should not be diagnosed when an active medical or psychiatric condition which may explain the fatigue is identified. We discuss a number of recent prospective studies that have provided valuable insights into the aetiology of chronic fatigue and describe a model for understanding chronic fatigue which may be equally relevant regardless of whether or not an apparent medical cause for fatigue can be identified.

See the associated research paper by Jones et al:

## Commentary

Medical students are often told that the sound of approaching hooves is more likely to herald the arrival of horses than zebras. The metaphor reinforces the idea that in medicine common things happen commonly and that clinicians should avoid spending too much time chasing rare or unlikely diagnoses. Fatigue is a very common clinical problem with many possible causes [1,2]. Some causes of fatigue are common 'horses' such as anaemia, viral infections, sleep deprivation, diabetes and depression. However, potential 'zebras' such as malignancy or auto-immune disorders may also present with fatigue. Even with extensive investigations, the underlying aetiology of an individual's fatigue in many cases remains unknown. Over recent decades there has been increasing recognition of a group of individuals with severe, persistent, and unexplained fatigue [3]. Such persistent fatigue has, at times, been seen as an illness of modern life, although there is good evidence to show that chronic fatigue has been a common problem since at least the 19th century, but under different diagnostic labels, such as neurasthenia [4,5]. Some, but by no means all, of these individuals fulfil the current criteria for chronic fatigue syndrome (CFS), which requires that persisting or relapsing fatigue be present for at least 6 months, is not relieved by rest, is not explained by medical or psychiatric conditions and is accompanied by a range of cognitive and somatic symptoms [6]. Here, we discuss a paper by Jones *et al*. published this month in *BMC Medicine *[7], as well as recent prospective studies that provide valuable insights into the aetiology and contribute to a model for understanding chronic fatigue.

At present, and despite much effort, there are no investigative tools or physical signs that can confirm the presence of CFS and it remains a diagnosis of exclusion [8]. As a result, clinicians must decide how long to keep looking for alternative explanations for fatigue before settling on a diagnosis of CFS. There are numerous cautionary tales of individuals who have suffered from delayed or missed diagnoses of serious illnesses due to under investigating of fatigue [9]. Yet if the search for unlikely 'zebra' causes of fatigue goes on too long, the risk of iatrogenic harm increases and the opportunity for early focused treatment of CFS may be lost [10].

Studies based in specialized clinics have suggested that yields from detailed investigations of those with prolonged fatigue are low, with only 5% of laboratory tests revealing an underlying cause [11]. However, fatigued patients seen in specialized clinics differ from those seen in other settings [12], with some reports suggesting higher yields from investigations may be possible in primary care [13]. The recently published VAMPIRE study based in Dutch primary care found that 8% of patients presenting with fatigue had a blood test detectable somatic illness diagnosed over a 1-year follow-up period, with the vast majority of the disorders identified from a very limited set of simple blood tests (haemoglobin, erythrocyte sedimentation rate, glucose and thyroid-stimulating hormone) [14]. The UK-based National Institute of Health and Clinical Excellence guidelines on the diagnosis and management of CFS recommend a slightly more conservative approach, with a more extensive list of blood and urine investigations suggested (Appendix) [8]. Such lists of physical investigations should not detract from the need to consider psychological causes of fatigue. Depression is very common amongst those with fatigue [4,15], with recent studies using the British birth cohorts showing over 70% of adults reporting CFS have evidence of psychiatric disorder prior to their fatigue symptoms beginning [16].

A clinician assessing a patient in the community with apparent CFS may well ask 'If I look, how likely am I to find a contributing medical or psychiatric cause for the fatigue, and what difference will this make?' A paper by Jones *et al*. [7] may help to answer these questions. Using random telephone surveys, 904 people who met the criteria for CFS were identified. On telephone history alone they were able to identify a potential cause of the fatigue in 441 (48%). When the remaining cases were seen for a physical examination, psychiatric interview and laboratory screening, potential medical or psychiatric causes of fatigue were identified in a further 49%. Not surprisingly, the most common co-morbid conditions identified were depression, followed by bipolar affective disorder, thyroid disease, substance misuse and diabetes. Obesity, already known to be associated with a number of these conditions [17-19], increased the chances of a medical or psychiatric cause being identified. These results are very similar to a Dutch study published earlier this year, which found concomitant diseases which could cause fatigue in 55.5% of those reporting chronic fatigue lasting more than 6 months [20]. Based on these findings, clinicians should feel encouraged that, if they look for common psychiatric and medical conditions in those complaining of prolonged fatigue, the rate of detection will be higher than previously thought. Thus, current recommendations advising a range of simple investigations (Appendix) for those with persistent fatigue seem well placed. Jones *et al*. did find some 'zebras' but, as expected, these were relatively rare. A simple mental state examination appears to remain the most productive single investigation in any new person presenting with unexplained fatigue [21].

**Figure 1:**
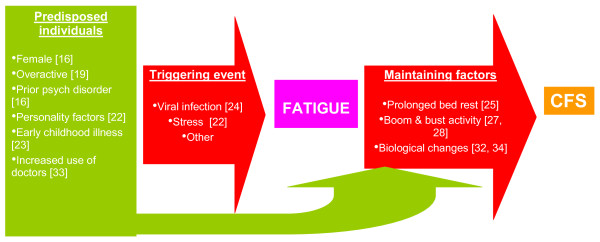
**Proposed model for understanding the aetiology of chronic fatigue syndrome (CFS) **[10,16,19,22-28,32-34].

The identification of potentially treatable causes of fatigue has obvious clinical importance. However, Jones *et al*.'s findings also raise questions about the nature of chronic fatigue. Currently, most diagnostic criteria suggest CFS should not be diagnosed when an active medical or psychiatric condition is identified which may explain the fatigue [6]. This implies that the aetiology of 'unexplained' CFS is different to that of the 'explained' fatigue seen in those with a diagnosed medical condition. While we are yet to fully understand the causes of CFS, a number of prospective studies have given us some knowledge of those at high risk, and which pathways to fatigue seem to be important [16,19,22-25]. Based on these findings, a model of the aetiology of CFS can be constructed, as demonstrated in Figure 1. This suggests that CFS results from a combination of pre-morbid risk, followed by an acute event leading to fatigue, and then a pattern of behavioural and biological responses contributing to a prolonged severe fatigue syndrome [26]. Based on this model, the initial cause of the fatigue has a limited impact on the eventual course of the illness. Rather, it is the maintaining factors, such as dramatic fluctuations in levels of activity (so called 'boom and bust' cycles), that need to be addressed if recovery is to occur [27,28]. This model has been developed from research focused on the 'unexplained' fatigue of CFS. However, there is emerging evidence which suggests that it may be appropriate to extend it to encompass fatigue with an apparent medical cause. There are numerous examples of studies demonstrating that the fatigue associated with clear 'physical' illnesses is more closely associated with behavioural and psychological factors than with the severity of the underlying illness. For example, fatigue in HIV-infected patients is more strongly associated with psychological factors than with measures of HIV disease progression or the use of highly active antiretroviral drugs [29]. There is also evidence that behaviourally focused interventions are some of the most effective ways of reducing fatigue, even when there is a clear underlying cause, such as rheumatoid arthritis, multiple sclerosis or cancer [30,31]. Thus, it may be that the divide between fatigue secondary to diagnosed medical problems and CFS may be need to be made more permeable, with some relaxing of the exclusion criteria in diagnostic guidelines for CFS. This may allow a greater use of evidence-based treatments developed for treating CFS amongst those with an apparent medical or psychiatric cause of their fatigue.

## Abbreviations

CFS: chronic fatigue syndrome.

## Competing interests

The authors declare that they have no competing interests.

## Authors' contributions

The authors contributed equally to the writing of this article

## Appendix

### Investigations recommended by the UK National Institute of Clinical Excellence to exclude medical causes of chronic fatigue ([8])

Tests that should usually be done:

• urinalysis for protein, blood and glucose

• full blood count

• urea and electrolytes

• liver function

• thyroid function

• erythrocyte sedimentation rate or plasma viscosity

• C-reactive protein

• random blood glucose

• serum creatinine

• screening blood tests for gluten sensitivity

• serum calcium

• creatine kinase

• assessment of serum ferritin levels (children and young people only).

Additional serology tests that should only be done if the history suggests the possibility of a recent infection:

• chronic bacterial infections, such as borreliosis

• chronic viral infections, such as HIV or hepatitis B or C

• acute viral infections, such as infectious mononucleosis (use heterophile antibody tests)

• latent infections, such as toxoplasmosis, Epstein-Barr virus or cytomegalovirus.

## Pre-publication history

The pre-publication history for this paper can be accessed here:



## References

[B1] Cox BD (1987). The health and lifestyle survey.

[B2] Rosenthal TC, Majeroni BA, Pretorius R, Malik K (2008). Fatigue: an overview. Am Fam Physician.

[B3] Wessely S, Hotopf M, Sharpe M (1998). Chronic Fatigue and its Syndromes.

[B4] Harvey SB, Wessely S, Kuh D, Hotopf M (2009). The relationship between fatigue and psychiatric disorders: Evidence for the concept of neurasthenia. J Psychosom Res.

[B5] Wessely S (1990). Old wine in new bottles: neurasthenia and 'ME'. Psychol Med.

[B6] Fukuda K, Straus SE, Hickie I, Sharpe MC, Dobbins JG, Komaroff A (1994). The chronic fatigue syndrome: a comprehensive approach to its definition and study. International Chronic Fatigue Syndrome Study Group. Ann Intern Med.

[B7] Jones J, Lin J, Maloney E, Boneva R, Nater U, Unger E, Reeves W (2009). An evaluation of exclusionary medical/psychiatric conditions in the definition of chronic fatigue syndrome. BMC Medicine.

[B8] NICE (2007). Chronic fatigue syndrome/myalgic encephalomylitis (or encephalopathy). Diagnosis and management of CFS/ME in adults and children.

[B9] Hurel SJ, Abuiasha B, Baylis PH, Harris PE (1995). Patients with a self diagnosis of myalgic encephalomyelitis. Bmj.

[B10] Harvey SB, Wessely S (2009). Tired all the time: can new research on fatigue help clinicians?. Br J Gen Pract.

[B11] Lane TJ, Matthews DA, Manu P (1990). The low yield of physical examinations and laboratory investigations of patients with chronic fatigue. Am J Med Sci.

[B12] Euba R, Chalder T, Deale A, Wessely S (1996). A comparison of the characteristics of chronic fatigue syndrome in primary and tertiary care. Br J Psychiatry.

[B13] Elnicki DM, Shockcor WT, Brick JE, Beynon D (1992). Evaluating the complaint of fatigue in primary care: diagnoses and outcomes. Am J Med.

[B14] Koch H, van Bokhoven MA, ter Riet G, van Alphen-Jager JT, Weijden T van der, Dinant GJ, Bindels PJ (2009). Ordering blood tests for patients with unexplained fatigue in general practice: what does it yield? Results of the VAMPIRE trial. Br J Gen Pract.

[B15] Nater UM, Lin JM, Maloney EM, Jones JF, Tian H, Boneva RS, Raison CL, Reeves WC, Heim C (2009). Psychiatric comorbidity in persons with chronic fatigue syndrome identified from the Georgia population. Psychosom Med.

[B16] Harvey SB, Wadsworth M, Wessely S, Hotopf M (2008). The relationship between prior psychiatric disorder and chronic fatigue: evidence from a national birth cohort study. Psychol Med.

[B17] Foresight (2007). Tackling Obesities: Future Choices - Project Report.

[B18] Rivenes AC, Harvey SB, Mykletun A (2009). The relationship between abdominal fat, obesity, and common mental disorders: results from the HUNT study. J Psychosom Res.

[B19] Harvey SB, Wadsworth M, Wessely S, Hotopf M (2008). Etiology of chronic fatigue syndrome: testing popular hypotheses using a national birth cohort study. Psychosom Med.

[B20] Van't Leven M, Zielhuis GA, Meer JW van der, Verbeek AL, Bleijenberg G (2009). Fatigue and chronic fatigue syndrome-like complaints in the general population. Eur J Public Health.

[B21] Manu P, Matthews DA, Lane TJ (1988). The mental health of patients with a chief complaint of chronic fatigue. A prospective evaluation and follow-up. Arch Intern Med.

[B22] Kato K, Sullivan PF, Evengard B, Pedersen NL (2006). Premorbid predictors of chronic fatigue. Archives of General Psychiatry.

[B23] Viner R, Hotopf M (2004). Childhood predictors of self reported chronic fatigue syndrome/myalgic encephalomyelitis in adults: national birth cohort study. Bmj.

[B24] White PD, Thomas JM, Amess J, Crawford DH, Grover SA, Kangro HO, Clare AW (1998). Incidence, risk and prognosis of acute and chronic fatigue syndromes and psychiatric disorders after glandular fever. Br J Psychiatry.

[B25] White PD, Thomas JM, Kangro HO, Bruce-Jones WD, Amess J, Crawford DH, Grover SA, Clare AW (2001). Predictions and associations of fatigue syndromes and mood disorders that occur after infectious mononucleosis. Lancet.

[B26] White PD (2004). What causes chronic fatigue syndrome?. Bmj.

[B27] Deale A, Chalder T, Marks I, Wessely S (1997). Cognitive behavior therapy for chronic fatigue syndrome: a randomized controlled trial. Am J Psychiatry.

[B28] Surawy C, Hackmann A, Hawton K, Sharpe M (1995). Chronic fatigue syndrome: a cognitive approach. Behav Res Ther.

[B29] Henderson M, Safa F, Easterbrook P, Hotopf M (2005). Fatigue among HIV-infected patients in the era of highly active antiretroviral therapy. HIV medicine.

[B30] Armes J, Chalder T, Addington-Hall J, Richardson A, Hotopf M (2007). A randomized controlled trial to evaluate the effectiveness of a brief, behaviorally oriented intervention for cancer-related fatigue. Cancer.

[B31] Neill J, Belan I, Ried K (2006). Effectiveness of non-pharmacological interventions for fatigue in adults with multiple sclerosis, rheumatoid arthritis, or systemic lupus erythematosus: a systematic review. J Adv Nurs.

[B32] Cleare AJ (2004). The HPA axis and the genesis of chronic fatigue syndrome. Trends Endocrinol Metab.

[B33] Hamilton WT, Hall GH, Round AP (2001). Frequency of attendance in general practice and symptoms before development of chronic fatigue syndrome: a case-control study. Br J Gen Pract.

[B34] Lyall M, Peakman M, Wessely S (2003). A systematic review and critical evaluation of the immunology of chronic fatigue syndrome. J Psychosom Res.

